# Functional Beverages From Frankincense and *Pistacia atlantica*: A Natural Synbiotic Strategy for Managing Hyperlipidemia and Modulating Gut Microbiota

**DOI:** 10.1002/fsn3.71075

**Published:** 2025-11-04

**Authors:** Nawfal Alhelfi, Zainab Tariq Yaseen Al‐Edany, Ammar B. Altemimi, Zheng Ruan, Rawaa H. Tlay, Tarek Gamal Abedelmaksoud

**Affiliations:** ^1^ Food Science Department, College of Agriculture University of Basrah Basrah Iraq; ^2^ State Key Laboratory of Food Science and Resources, Institute of Nutrition Nanchang University Nanchang China; ^3^ Department of Food Science, College of Agricultural Engineering Damascus University Damascus Syria; ^4^ Food Science Department, Faculty of Agriculture Cairo University Giza Egypt

**Keywords:** *Boswellia serrata*, gut microbiota, hypercholesterolemia, lipid metabolism, *Pistacia atlantica*, synbiotic beverages

## Abstract

Functional beverages enriched with *Boswellia serrata* (frankincense) and *Pistacia atlantica* , alone or combined with probiotics ( *Lactobacillus acidophilus* ) and prebiotics (fructooligosaccharides, FOS), were evaluated for their impact on gut microbiota and lipid metabolism in hypercholesterolemic rats over 45 days. The beverages significantly modulated fecal microbiota by increasing lactic acid bacteria (LAB) and reducing *Escherichia coli* and coliform counts. The synbiotic formulation containing *Pistacia atlantica* , probiotics, and prebiotics produced the most notable effects, achieving the highest LAB counts (log 7.51 cfu/g) and the lowest *E. coli* (log 3.72 cfu/g) and coliform counts (log 4.02 cfu/g). This formulation also led to marked reductions in serum triglycerides, total cholesterol, and very low‐density lipoprotein (VLDL), while elevating high‐density lipoprotein (HDL) levels. Improved probiotic viability during 28‐day refrigerated storage further highlighted the protective role of prebiotics. These outcomes are attributed to the synergistic effects of phenolic and terpenoid compounds (e.g., α‐pinene, limonene) and gut microbiota‐mediated mechanisms, including short‐chain fatty acid production. Overall, synbiotic beverages incorporating *Pistacia atlantica* and *Boswellia serrata* show strong potential as functional dietary interventions to enhance gut health, reduce hypercholesterolemia, and extend probiotic shelf life, offering promising applications in the development of natural therapeutic beverages.

## Graphical abstract

Functional beverages enriched with *Boswellia serrata* (frankincense) and *Pistacia atlantica* , combined with probiotics and prebiotics, improved gut microbiota balance and lipid profiles in hypercholesterolemic rats. The synbiotic *Pistacia atlantica* formulation achieved the greatest effects, increasing lactic acid bacteria while reducing *E. coli* , coliforms, triglycerides, and cholesterol, and enhancing HDL levels. These benefits, supported by phenolic–terpenoid compounds and microbiota‐mediated mechanisms, underscore the potential of synbiotic beverages as natural therapeutic interventions for gut health and the management of hypercholesterolemia.

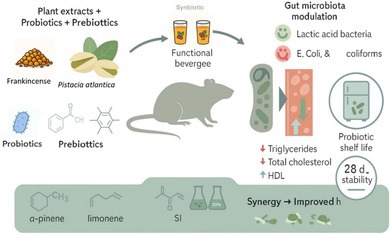

## 1 Introduction

Hyperlipidemia, defined by elevated cholesterol and triglycerides, is a major global health burden and a leading contributor to cardiovascular diseases (CVDs), projected to cause over 23 million deaths by 2030 (Nawsherwan et al. 2022). While lipid‐lowering therapies such as statins remain the standard, their limited efficacy, side effects, and poor adherence highlight the need for alternative strategies (Vekic et al. 2022). Recent advances emphasize the pivotal role of the gut microbiota in regulating lipid metabolism and inflammation associated with CVD (Yang et al. 2021). Dysbiosis has been linked to obesity, insulin resistance, and hyperlipidemia (Li et al. 2022). Nutritional interventions targeting gut microbes, including probiotics, prebiotics, and synbiotics, show promise in modulating lipid profiles. Probiotics like Lactobacillus spp. can lower serum cholesterol and triglycerides through bile salt hydrolase activity, cholesterol assimilation, and short‐chain fatty acid production (Ooi and Liong 2010). Prebiotics further support beneficial microbial growth, and synbiotics offer synergistic effects (Esmail et al. 2022). In parallel, bioactive compounds from medicinal plants are gaining attention for their lipid‐lowering, antioxidant, and anti‐inflammatory effects. Boswellia and *Pistacia atlantica* , rich in terpenoids, phenolics, and essential oils, have long been used in traditional medicine for cardiovascular disorders, making them promising complementary therapies (Buyel 2018).

Frankincense, derived mainly from *Boswellia sacra* , has long been valued for its therapeutic and cultural roles, largely due to its bioactive boswellic acids. These compounds exert strong anti‐inflammatory and antioxidant effects, particularly through inhibition of 5‐lipoxygenase, thereby reducing leukotriene‐mediated inflammation linked to atherosclerosis and other chronic disorders (DeCarlo et al. 2022; Hussain et al. 2022). Beyond its traditional use in inflammatory conditions, frankincense has also been investigated for its role in managing metabolic syndrome, including dyslipidemia and diabetes, highlighting its broader cardiometabolic relevance (Al‐Daraji et al. 2013; Ahangarpour et al. 2014). Similarly, *Pistacia atlantica* , native to Mediterranean and Middle Eastern regions, possesses a rich phytochemical composition encompassing phenolics, flavonoids, and essential fatty acids with antioxidant, anti‐inflammatory, and lipid‐regulating activities (Elyasi Ghahfarrokhi et al. 2022; Bakka et al. 2022). Traditionally known as “Baneh,” it has been widely used in Iranian and Iraqi medicine for metabolic disorders (Ahmed 2017). Experimental studies confirm its ability to lower LDL‐C, total cholesterol, and triglycerides while enhancing HDL‐C levels in hyperlipidemic models, with additional protective effects on vascular and hepatic tissues (Ghalavand et al. 2022). Together, frankincense and *Pistacia atlantica* represent promising natural interventions with complementary antioxidant, anti‐inflammatory, and lipid‐modulating properties, supporting their potential as adjunct therapies for cardiovascular and metabolic health.

Despite the promising individual benefits of frankincense and *Pistacia atlantica* , there is a paucity of research investigating their combined effects, particularly when integrated with synbiotic components to modulate gut microbiota and enhance cardiovascular health. Given the multifactorial nature of hyperlipidemia involving lipid metabolism, inflammation, and gut microbial interactions, a synergistic approach combining bioactive phytochemicals with probiotics and prebiotics could provide additive or even synergistic benefits. This study therefore aims to develop and evaluate a novel functional beverage enriched with male frankincense and *Pistacia atlantica* extracts, fortified with synbiotic elements. The objective is to assess its potential in improving lipid profiles by increasing HDL cholesterol while reducing total cholesterol, LDL cholesterol, and triglycerides through the dual action of bioactive compounds and microbiota modulation. This integrative strategy addresses a critical research gap and aligns with the growing interest in natural, multi‐targeted, and microbiota‐focused interventions for cardiovascular disease prevention.

## 2 Materials and Methods

### 2.1 Materials

*Pistacia atlantica* subsp. kurdica fruits were obtained from the Kurdistan Region of Iraq, while *Boswellia sacra* gum resin was sourced from a local market in Basra, Iraq. The botanical identity of the gum resin was confirmed by specialists at the Department of Plant Protection, College of Agriculture, University of Basra.

### 2.2 Chemicals

Analytical‐grade chemicals and reagents were used throughout the study. Gallic acid and quercetin standards were purchased from BDH Chemicals (England), while the Folin–Ciocalteu reagent was obtained from Avonchem (UK). Sodium carbonate was sourced from Merck (Germany), sodium nitrite and potassium ferricyanide from CDH (India), and sodium hydroxide from VWR Chemicals (USA). Aluminum chloride and ferric chloride were supplied by BDH (England), and methanol was obtained from SRL (India). The free radical compound 2,2‐diphenyl‐1‐picrylhydrazyl (DPPH) was purchased from Sigma‐Aldrich (Germany). Ascorbic acid was procured from HiMedia (India), and trichloroacetic acid was also sourced from CDH (India). Cholesterol powder was obtained from Reagent World (USA), and diethyl ether was supplied by BDH (England). The study also utilized high‐purity (≥ 99%) standards of several phenolic compounds including rutin, gallic acid, apigenin, epicatechin, catechin, kaempferol, and quercetin, all purchased from Sigma‐Aldrich (Germany).

### 2.3 Instrumentation

Instrumentation included a benchtop centrifuge (GEMMY, Taiwan) and a UV–Visible spectrophotometer (Optima, England), which were employed for sample preparation and absorbance measurements, respectively.

### 2.4 Preparation for the Aqueous Extract of Frankincense and *Pistacia atlantica*

The collected gum exudate, either in lump or granulated form, was first pulverized into a fine powder. Subsequently, 50 g of the resulting powder were dissolved in 500 mL of distilled water. The solution was allowed to hydrate and extract at ambient temperature (approximately 25°C) for 24 h to facilitate maximum solubilization of soluble gum constituents. Following extraction, the solution was transferred into 15‐mL Falcon centrifuge tubes and centrifuged at 1000 rpm for 10 min to separate insoluble residues. The resulting supernatants were carefully decanted and subjected to vacuum filtration using a Buchner funnel equipped with Whatman No. 15 filter paper to remove any remaining particulates. The clarified filtrates were subsequently stored at 4°C until further processing. For extract preservation and concentration, the samples were lyophilized using a laboratory freeze‐drying system as described by Khalaj‐Kondori et al. (2016), yielding powdered gum extracts suitable for downstream applications.

### 2.5 Antioxidant Activities for the Aqueous Extract of Frankincense and *Pistacia atlantica*

#### 2.5.1 DPPH Radical‐Scavenging Activity

The antioxidant activity was assessed using the DPPH radical scavenging assay following the procedure described by Phuyal et al. (2020). Briefly, different concentrations of the sample solutions were prepared, and 1 mL of each was mixed with 1 mL of DPPH solution. The mixtures were incubated at room temperature for 30 min in the dark. A control was prepared by mixing 1 mL of methanol with 1 mL of DPPH solution. Absorbance was measured at 517 nm using a UV–Vis spectrophotometer, with ascorbic acid serving as the standard reference. The radical scavenging activity was expressed as the percentage of inhibition, calculated using the following equation: R % = (AC−AS)/AC × 100. The AC absorbance of the control (consisting of 1 mL methanol and 1 mL DPPH solution), the AS absorbance of the sample solution, and R% representing the degree of radical scavenging activity were recorded.

#### 2.5.2 Ferric Reducing Antioxidant Power (FRAP) Activity

The Ferric Reducing Antioxidant Power (FRAP) assay evaluates the capacity of a substance to reduce ferric ions (Fe^3+^) to ferrous ions (Fe^2+^), thereby indicating its antioxidant potential. For the assay, a test tube was filled with 2 mL of sodium phosphate buffer (0.2 M, pH 6.6), 2 mL of 1% potassium ferricyanide, and varying concentrations of the aqueous extract (500 and 1000 μg/mL). The mixture was incubated in a water bath at 50°C for 20 min. To terminate the reaction, 2 mL of 10% trichloroacetic acid was added, followed by centrifugation at 3000 rpm for 10 min. Subsequently, 2 mL of the supernatant was mixed with 2 mL of distilled water and 0.5 mL of freshly prepared 1% ferric chloride (FeCl₃) solution. The absorbance of the resulting solution was measured at 700 nm using a spectrophotometer. Ascorbic acid served as the positive control, and the antioxidant capacity was expressed based on the absorbance values obtained (Védékoi et al. 2019).

### 2.6 Determination of Phenolic Components in Frankincense and *Pistacia atlantica* by HPLC

To get the phenolic compounds out of the homogenized plant, 100 mL of chloroform was added to 20 g of the plant after it had been ground up well and put on an electric vibrator for 3 h to get rid of the fat. The chloroform layer was then removed, and the sample was dried at 50°C to make sure there were no chloroform residues left. Then, a 70/30 mix of ethanol and water was used to extract 10 g of the dried sample. An ultrasonic bath (USA) was used for the extraction process, which lasted for 1 h at room temperature. 5 mL of the liquid extract that had been filtered was used to figure out the extraction yield. Using a rotating evaporator in a vacuum (Slovenia), the solvent was taken away, and the mixture was dried at 40°C until the mass stayed the same. To keep the dry samples from going bad before they were analyzed, they were kept in glass bottles at 4°C. Different amounts of phenolic substances were measured using reversed‐phase HPLC analysis on a SYKAMN HPLC chromatographic machine with a UV detector (Chemstation). A Zorbax Eclipse Plus‐C18‐OSD, 25 cm, 4.6 mm column. The temperature of the column was 30°C. This is how the gradient elution method was done: eluent A was methanol, and eluent B was 1% formic acid in water (v/v) as follows: Initial 0–4 min, 40% B; 4–10 min, 50% B; and flow rate of 0.7 mL/min. The samples and standards were injected with a volume of 100 μL each, utilizing an autosampler for automated injection. The spectra were obtained at a wavelength of 280 nm (Radovanović et al. 2015).

### 2.7 Gas Chromatography–Mass Spectrometry Analysis (GC–MS) for the Essential Oils of Frankincense and *Pistacia atlantica* Gum

The following settings were used to perform GC–MS analysis on a GC‐mass 5977A Series Agilent system auto sampler and gas chromatograph interfaced to a mass spectrometer (GC–MS) instrument: Column HP‐5MS (30 × 0.25 mm I.D.) fused silica capillary column Elite‐1 working in electron impact mode at 70 eV; helium (99.999%) was utilized as the carrier gas, with a steady flow of 1 mL/min and an injection volume of 0.5 uL (split ratio of 10:1), at a temperature of 250°C for the injector and 280°C for the ion source. The oven was set to start at 60°C (isothermal for 2 min), climb by 10°C/min to 270°C, then increase by 5°C/min to 290°C, and conclude with a 9 min isothermal at 310°C. Mass spectra were obtained at 70 eV with pieces ranging from 45 to 450 Da and a scan interval of 0.5 s. The GC takes 60 min to complete (Eswaran et al. 2012).

### 2.8 Preparation of Functional Beverages From Frankincense and *Pistacia atlantica*

The raw beverages were prepared by dissolving the gum in water at a ratio of 1:100 (w/v). Following a 24 h soaking period, the mixture was filtered to remove any residual gum particles. The resulting filtrate was then aseptically transferred into sterile glass containers and subjected to pasteurization at 85°C for 15 min (Manjula et al. 2021) before refrigeration. These raw beverages served as the base for the formulation of functional beverages. Subsequently, the raw beverage was aliquoted into sterile glass containers, to which fructo‐oligosaccharide (FOS) was incorporated as a prebiotic at concentrations ranging from 0% to 1% (w/v), following the method of Nguyen et al. (2019). The containers underwent a second pasteurization step under the same conditions (85°C for 15 min) (Manjula et al. 2021). A 1 mL dosage was selected based on preliminary studies demonstrating optimal therapeutic efficacy and cost‐effectiveness. Moreover, the 1% (w/v) FOS concentration was identified as optimal for enhancing probiotic viability, thereby supporting the development of a functional health beverage with potential for industrial‐scale production.

**Activation of probiotic bacteria**, the lyophilized *Lactobacillus acidophilus* La‐5 culture was activated through three successive cycles of incubation in MRS broth, following the protocol described by Mokhtari et al. (2019). Briefly, the bacterial suspension was incubated anaerobically at 37°C for 24 h. Subsequently, the cultures were centrifuged to separate the bacterial cells, and the MRS broth supernatant was carefully decanted. The bacterial pellet was then resuspended in sterile normal saline and vortexed to achieve a turbidity equivalent to the McFarland standard, corresponding to an approximate bacterial concentration of 10^8^ CFU/mL (Xu et al. 2020). The optical density of the suspension was measured at 600 nm using a spectrophotometer, which was previously zeroed with normal saline as the blank.

**Inoculate functional beverages with probiotic bacteria**, probiotic bacteria *Lactobacillus acidophilus* La‐5 was aseptically inoculated into the beverages at a concentration of 1% (v/v) inside a Biological Safety Cabinet under sterile conditions (Hossain et al. 2020). The inoculum concentration was standardized at 10^8^ CFU/mL (Xu et al. 2020). Subsequently, the functional beverage samples were incubated at 37°C for 24 h to initiate the fermentation process. Following fermentation, the samples were stored at refrigeration temperature for preservation.

### 2.9 Animal Experiment

Adult male *Rattus norvegicus* rats were obtained from Anbar, Iraq, weighing 170–190 g and 4 months old, were employed in the investigation. At the College of Veterinary Medicine/University of Basra animal house, 36 rats were divided into 6 groups of 6 rats. Clean plastic cages held them. Rats lived in a well‐ventilated room at 24°C–28°C and 50%–55% humidity. The illumination was 12 h natural light and 12 h darkness. Clean water and healthy food were provided, and they could have them anytime they wished. The induction period was 45 days. Table 1 presents the composition of the diets formulated for the negative and positive control groups used in the hyperlipidemia model. The negative control feed was prepared without the addition of cholesterol, while the positive control feed included 1% cholesterol to induce hyperlipidemia. Both diets consisted of standard feed ingredients such as skim milk, corn starch, vitamins and minerals, corn oil, and cellulose, with the only difference being the inclusion of cholesterol in the positive control feed.

**TABLE 1:** Nutritional composition of standard feed for negative and positive control groups in a hyperlipidemia model (with and without cholesterol supplementation).

Ingredients (g)	Negative control feed	Positive control feed
Skim milk	200	200
Corn starch	670	660
Vitamins and minerals	30	30
Corn oil	50	50
Cellulose	50	50
Cholesterol	0	10

#### 2.9.1 Experimental Design

The experimental treatments were conducted in the following manner: **T1 (Negative control) (C‐)** The animals were fed a standard diet throughout the experiment of 45 days +water. **T2 (positive control) (C+)** The animals were fed a standard diet with 1% cholesterol added throughout the experiment of 45 days +water. **T3 Frankincense (F)** The animals were fed a standard diet with 1% cholesterol added and took a daily dose of 1 mL of the raw beverage of frankincense throughout the experiment of 45 days +water. **T4 Frankincense + Synbiotics (F S)** The animals were fed a standard diet with 1% cholesterol added and took a daily dose of 1 mL of the functional beverage of frankincense with Synbiotics ( *Lactobacillus acidophilus* + FOS) throughout the experiment of 45 days +water. **T5 *Pistacia atlantica* (P.A)** The animals were fed a standard diet with 1% cholesterol added and took a daily dose of 1 mL of the raw beverage of *Pistacia atlantica* throughout the experiment of 45 days +water. **T6 *Pistacia atlantica* + Synbiotics (P.A S)** The animals were fed a standard diet with 1% cholesterol added and took a daily dose of 1 mL of the functional beverage of *Pistacia atlantica* with Synbiotics ( *Lactobacillus acidophilus* + FOS) throughout the experiment of 45 days +water.

#### 2.9.2 Dosing Method

The experimental animals were dosed daily throughout the 45‐day trial period with 1 mL using an oral gavage needle.

#### 2.9.3 Collection of Samples

Feces collection, Fecal samples were collected aseptically in tightly sealed plastic containers and promptly transported to the laboratory for microbial enumeration analysis. For blood sample collection, rats were fasted for approximately 12 h before anesthesia induction using diethyl ether administered via cotton soaked with the agent. Each rat was placed inside a sealed glass jar to allow adequate inhalation of the anesthetic vapor. Once anesthetized, the animal was removed from the jar, positioned on the laboratory bench, and cardiac puncture was performed using a 5 mL sterile syringe to withdraw blood. The collected blood was transferred into gel separator tubes and centrifuged at 2000 rpm for 15 min to separate the serum. The serum was then aliquoted into Eppendorf tubes and stored at appropriate freezing temperatures until subsequent analyses were performed (Abass et al. 2016).

#### 2.9.4 Estimation of the Microbial Content of Rat Feces

An enumeration of *Lactobacillus* spp. was performed at the onset (time zero) and conclusion of the experiment using de Man, Rogosa and Sharpe (MRS) agar medium. For this purpose, 1 g of rat feces was collected and subjected to a series of decimal dilutions as described by Rios‐Covian et al. (2020). Subsequently, the diluted samples were inoculated via the pour plate method and incubated anaerobically at 37°C for 24 to 28 h. Coliform bacteria were enumerated using MacConkey agar medium, with incubation conducted aerobically at 37°C for 18 to 24 h. Additionally, *Escherichia coli* enumeration was carried out employing petri film methodology, following the procedure outlined by Bird et al. (2020).

#### 2.9.5 Biochemical Tests

Biochemical blood parameters, specifically lipid profile components including Total Cholesterol (TC), Low‐Density Lipoprotein (LDL), High‐Density Lipoprotein (HDL), Very Low‐Density Lipoprotein (VLDL), and Triglycerides (TG), were quantified in the serum of male rats administered raw and functional beverages. These analyses were conducted at the Elia Laboratory for Pathological Analysis in Basra, Iraq, utilizing a Mindray BS‐230 automated analyzer in accordance with the manufacturer's protocols.

### 2.10 Statistical Analysis

The statistical analysis was conducted using a completely randomized design (CRD) with a one‐factor experiment. Data analysis was performed employing the GenStat statistical software (Payne 2009). The evaluated factors were compared using the least significant difference (L.S.D.) test at a significance level of 0.05.

## 3 Results and Discussion

### 3.1 Antioxidant Activity of the Aqueous Extract of Frankincense and *Pistacia atlantica*

#### 3.1.1 DPPH Radical Scavenging Activity

**FIGURE 1:**
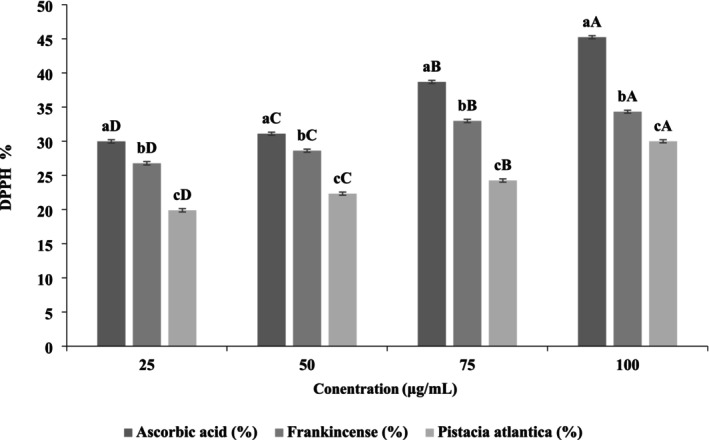
DPPH radical scavenging activity (%) of the aqueous extract of frankincense and *Pistacia atlantica* at different concentrations compared with ascorbic acid. The lowercase letters indicate significant differences between samples at the same concentration, while the uppercase letters denote significant differences between different concentrations of the same sample.

The antioxidant potential of the aqueous extracts of frankincense and *Pistacia atlantica* was evaluated using the DPPH radical scavenging assay. As shown in Figure 1, both extracts exhibited a concentration‐dependent increase in scavenging activity (*p* < 0.05). At all tested concentrations (25–100 μg/mL), frankincense extract demonstrated significantly higher activity than *Pistacia atlantica* (*p* < 0.05), although both remained lower than the ascorbic acid control. For instance, at 100 μg/mL, frankincense achieved 34.33% inhibition, compared with 30.00% for *Pistacia atlantica* and 45.12% for ascorbic acid. The statistical groupings (different lowercase letters within each concentration) confirm significant differences among samples, while uppercase letters indicate significant increases within each extract as the concentration increased (*p* < 0.05).

Mechanistically, the radical scavenging ability of these extracts can be attributed to their abundant phytochemicals, particularly polyphenols, terpenoids, and flavonoids. Compounds such as boswellic acids in frankincense and phenolic acids, tannins, and terpenes in *Pistacia atlantica* act as electron or hydrogen atom donors, stabilizing DPPH free radicals by converting them into non‐reactive species. Additionally, the presence of hydroxyl groups in these compounds enhances their capacity to delocalize unpaired electrons, thereby reducing oxidative stress (Obiștioiu et al. 2023; Hashemi et al. 2017).

The observed activity of frankincense in this study (26.83%–34.33%) aligns with previous reports showing strong dose‐dependent antioxidant responses, particularly when ethanolic extracts were used, achieving up to 86.44% inhibition at 100 μg/mL (Obiștioiu et al. 2023). Similarly, *Pistacia atlantica* displayed moderate but significant antioxidant potential, which is consistent with findings by Khoramdareh et al. (2022), who reported progressive increases up to 88.24% inhibition at higher concentrations (4 mg/mL). These differences emphasize the role of extraction solvent and phytochemical profile in determining radical scavenging efficiency.

Overall, the results confirm that both frankincense and *Pistacia atlantica* exhibit statistically significant, concentration‐dependent antioxidant activity. Their mechanisms involve direct radical scavenging through electron transfer and hydrogen atom donation, supported by their rich secondary metabolite composition. The findings underscore their potential as natural antioxidants, although their efficacy is influenced by extraction conditions and concentration compared to the standard ascorbic acid.

#### 3.1.2 Ferric Reducing/Antioxidant Power (FRAP)

**FIGURE 2:**
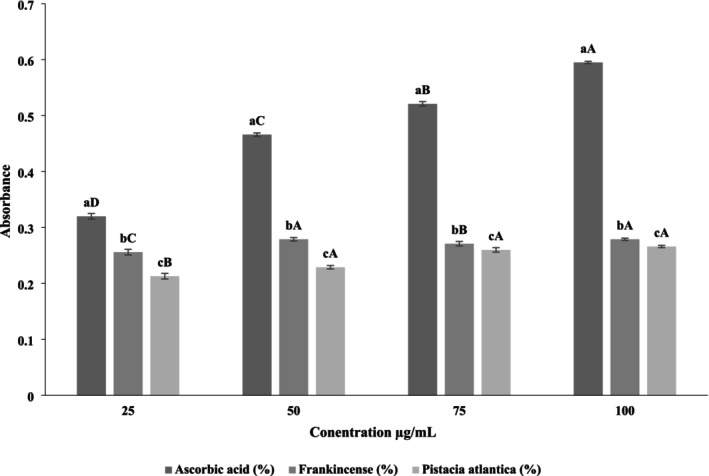
Ferric reducing antioxidant power (FRAP) of aqueous extracts of frankincense and *Pistacia atlantica* at different concentrations compared with ascorbic acid. The lowercase letters indicate significant differences between samples at the same concentration, while the uppercase letters denote significant differences between different concentrations of the same sample.

The FRAP assay evaluates the capacity of antioxidants to reduce ferric (Fe^3+^) to ferrous (Fe^2+^) ions via electron donation. The resulting Fe^2+^ forms a stable ferrous–tripyridyltriazine (Fe^2+^–TPTZ) complex that exhibits a strong blue coloration, with the absorbance intensity directly reflecting the reducing strength of the tested extract (Védékoi et al. 2019). As presented in Figure 2, both frankincense and *Pistacia atlantica* aqueous extracts demonstrated a clear concentration‐dependent increase in reducing power (*p* < 0.05). At all tested concentrations, ascorbic acid displayed the highest absorbance values, serving as the positive control. Frankincense consistently exhibited stronger reducing capacity than *Pistacia atlantica* (*p* < 0.05), with maximum absorbance values of 0.600 at 100 μg/mL compared with 0.281 for *Pistacia atlantica* . Statistical analysis indicated significant differences among the three samples at each concentration (different lowercase letters), and within each extract across concentrations (different uppercase letters), confirming a dose‐dependent enhancement of reducing activity (*p* < 0.05). Mechanistically, the ferric reducing potential of these extracts can be attributed to their abundant polyphenolic and terpenoid constituents, which act as effective electron donors. Hydroxyl groups in phenolic compounds facilitate electron transfer to ferric ions, stabilizing them as ferrous ions, while boswellic acids and tannins provide additional reducing sites. This mechanism not only strengthens the antioxidant capacity but also interrupts radical chain reactions by terminating reactive intermediates. The results obtained align with previous studies. Jha et al. (2020) observed a dose‐dependent increase in the FRAP activity of *Boswellia serrata* ethanolic extracts, with absorbance values ranging from 0.095 at 20 μg/mL to 0.312 at 100 μg/mL. Similarly, ethanolic and methanolic extracts of *Pistacia* species have been reported to exhibit substantial reducing power, although generally lower than synthetic standards such as ascorbic acid (Hashemi et al. 2017). These variations highlight the role of solvent polarity and phytochemical diversity in determining reducing capacity. In summary, both frankincense and *Pistacia atlantica* exhibited statistically significant and concentration‐dependent ferric reducing power, with frankincense showing greater efficacy. The findings confirm that their antioxidant activity operates primarily through electron transfer mechanisms, supporting their potential application as natural antioxidants in food and pharmaceutical formulations.

### 3.2 Phenolic Fractions in Frankincense and *Pistacia atlantica* by HPLC

**FIGURE 3:**
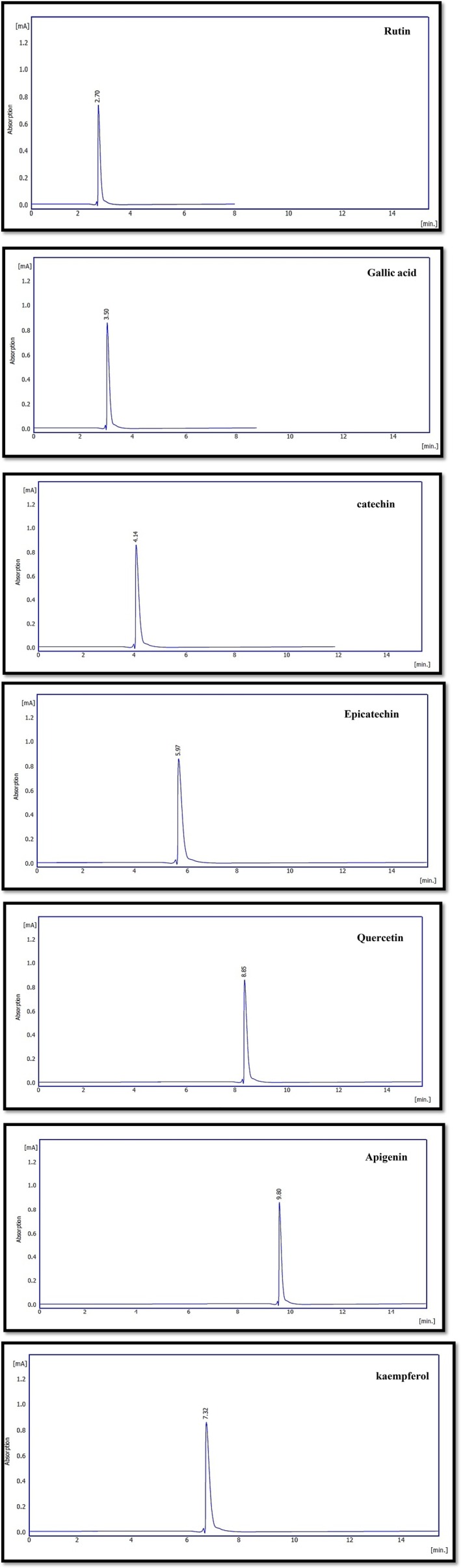
Standard phenolic compounds chromatogram in HPLC analysis.

**FIGURE 4:**
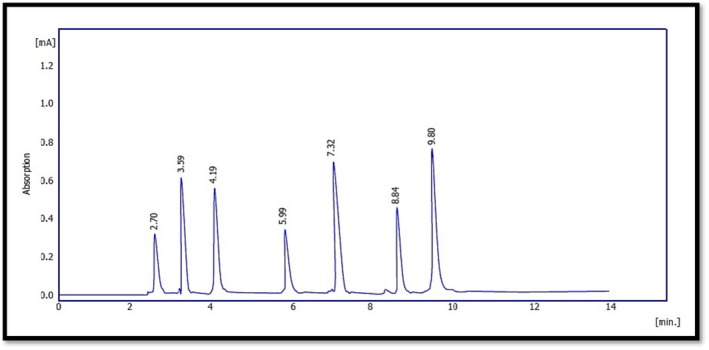
HPLC chromatogram of phenolic compounds in Frankincense.

**FIGURE 5:**
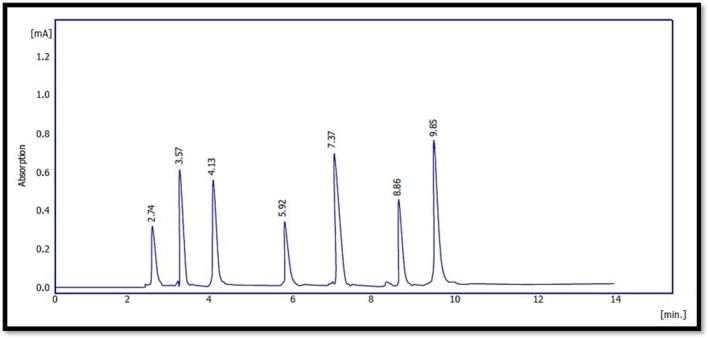
HPLC chromatogram of phenolic compounds in *Pistacia atlantica*.

The HPLC analysis revealed clear differences in the phenolic profiles of frankincense ( *Boswellia serrata* ) and *Pistacia atlantica* , as shown in Figures 3, 4, 5. Overall, gallic acid was the most dominant compound in frankincense (41.2%), followed by apigenin, kaempferol, catechin, and quercetin. In contrast, *P. atlantica* exhibited a broader diversity of phenolics, particularly flavonoids and tannins, consistent with previous reports (Ahmed et al. 2021). Mechanistically, these variations may be attributed to differences in the biosynthetic pathways of secondary metabolites across plant species. Frankincense resin is rich in phenolic acids due to the shikimate pathway, whereas *P. atlantica* accumulates higher levels of flavonoids and tannins, which play protective roles against oxidative stress and herbivory (Bozorgi et al. 2013; Rahman 2018). The predominance of gallic acid in both species highlights its central role as a precursor in hydrolyzable tannin biosynthesis, which may explain the strong antioxidant activity reported in these plants. Quantitative analysis (Tables 2, 3, 4) showed significant variation in compound concentrations between the two species. Such findings align with Sultan (2020), who reported free phenolic acids in frankincense resin, and with Ahmad et al. (2023), who confirmed the presence of quercetin, rutin, and catechin in *P. atlantica* leaves. The overlap in certain compounds (e.g., quercetin and catechin) suggests potential shared defense mechanisms and evolutionary conservation of phenolic metabolism, while the differences in relative abundances underline species‐specific adaptations. These results have important pharmacological implications. For instance, quercetin and catechin are potent antioxidants with documented anti‐inflammatory and antimicrobial effects, while apigenin and kaempferol contribute to anti‐cancer and cardioprotective properties (Rahman 2018). The high proportion of gallic acid, particularly in frankincense, could be directly linked to its strong radical scavenging ability, explaining part of its traditional medicinal value. Finally, the comparative HPLC profiling demonstrated that both frankincense and *P. atlantica* are rich sources of bioactive phenolics, but they differ in compound composition and relative abundance. These differences can be mechanistically explained by species‐specific metabolic pathways and ecological adaptations. Moreover, the overlap in key compounds reinforces their common pharmacological potential, supporting their use in traditional and modern medicine.

**TABLE 2:** Retention times and chromatographic characteristics of standard phenolic compounds.

Standard compounds chromatography							
No	Rt min	Area [mAU.s]	Height [mAU]	Area [%]	Height [%]	W 05	Compound name
1	2.7	1845	788.59	100	100	0.25	Rutin
2	3.5	2478.98	840.11	100	100	0.25	Gallic acid
3	4.14	1475	830.49	100	100	0.25	Catechin
4	5.97	1358.7	811.4	100	100	0.25	Epicatechin
5	7.32	1874	830.25	100	100	0.25	Kaempferol
6	8.85	2144.96	840.57	100	100	0.25	Quercetin
7	9.8	1897.49	851.48	100	100	0.25	Apigenin

**TABLE 3:** HPLC chromatographic characteristics of phenolic compounds identified in Frankincense.

Frankincense compounds chromatography								
No	Con. ppm	R_t_ min	Height [mAU]	Area [mAU]	Area [%]	Height [%]	W05[min]	Compound name
1	12.58	2.70	25148.97	350.12	11.00	11.00	0.10	Rutin
2	25.98	3.59	40112.65	580.44	14.00	14.00	0.15	Gallic acid
3	17.44	4.19	33985.47	851.49	13.00	13.00	0.15	Catechin
4	13.65	5.99	20651.44	360.99	11.00	11.00	0.10	Epicatechin
5	9.85	7.32	80652.44	580.14	19.00	19.00	0.20	Kaempferol
6	11.49	8.84	70445.19	530.69	13.00	13.00	0.10	Quercetin
7	16.25	9.80	97521.53	780.41	19.00	19.00	0.20	Apigenin
		Total	368517.49	4034.19	100.00	100.00		

**TABLE 4:** HPLC chromatographic characteristics of phenolic compounds identified in *Pistacia atlantica*.

*Pistacia atlantica* compounds chromatography								
No	Con. ppm	R_t_ min	Height [mAU]	Area [mAU]	Area [%]	Height [%]	W05[min]	Compound name
1	9.85	2.74	20251.49	341.45	11.00	11.00	0.10	Rutin
2	18.49	3.57	33652.08	574.11	14.00	14.00	0.15	Gallic acid
3	12.55	4.13	30985.14	841.59	13.00	13.00	0.15	Catechin
4	9.85	5.92	18552.41	352.99	11.00	11.00	0.10	Epicatechin
5	6.98	7.37	70652.31	576.54	19.00	19.00	0.20	Kaempferol
6	8.77	8.86	63963.65	524.59	13.00	13.00	0.10	Quercetin
7	13.25	9.85	90258.79	774.11	19.00	19.00	0.20	Apigenin
		Total	274858.99	4010.45	100.00	100.00		

### 3.3 Comparative Chemical Profiling of Essential Oils Extracted From Frankincense ( *Boswellia serrata* ) and *Pistacia atlantica* Gums Using GC–MS Analysis

**FIGURE 6:**
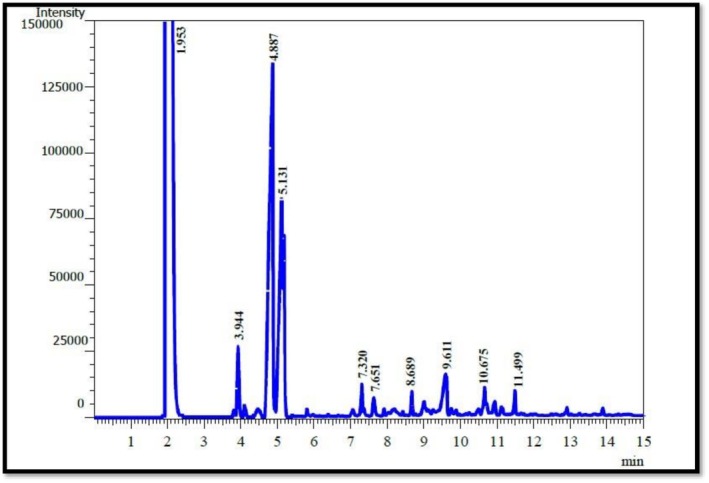
GC–MS chromatogram of frankincense essential oil.

**FIGURE 7:**
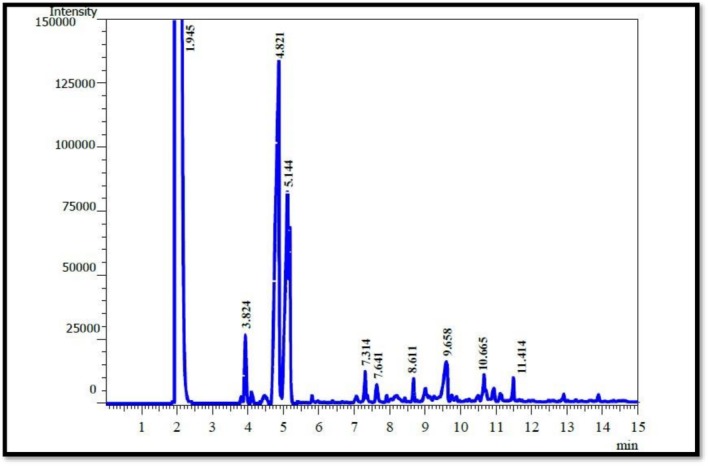
GC–MS chromatogram of *Pistacia atlantica* essential oil.

Gas chromatography–mass spectrometry (GC–MS) analysis revealed the presence of various volatile compounds in the essential oils extracted from frankincense ( *Boswellia serrata* ) and *Pistacia atlantica* gums. A total of nine major compounds were identified, accounting for 70.91% of the total oil composition in frankincense and 54.37% in *Pistacia atlantica* (Tables 5 and 6; Figures 6 and 7).

**TABLE 5:** Volatile chemical compounds of frankincense essential oil identified by GC–MS analysis.

Peak	Compounds	Ret. time	Con %
1	α‐pinene	3.944	8.55
2	β‐pinene	4.887	18.58
3	Limonene	5.131	16.55
4	P‐Cymene	7.32	6.25
5	Linalool	7.651	4.21
6	α‐Thujene	8.689	5.88
7	Camphene	9.611	4.25
8	Terpinene	10.675	2.65
9	Caryophyllene	11.499	3.99
Total			70.91%

**TABLE 6:** Volatile chemical compounds of *Pistacia atlantica* essential oil identified by GC–MS analysis.

No.	Compounds	Ret. time	Con %
1	α‐pinene	3.824	7.41
2	β‐pinene	4.821	13.66
3	Limonene	5.144	11.48
4	P‐Cymene	7.314	4.85
5	Linalool	7.641	3.99
6	α‐Thujene	8.611	3.98
7	Camphene	9.658	3.85
8	Terpinene	10.666	2.04
9	Caryophyllene	11.414	3.11
Total			54.37%

In frankincense gum essential oil, β‐pinene was the most abundant component (18.58%, RT = 4.887 min), followed by limonene (16.55%, RT = 5.131 min), α‐pinene (8.55%, RT = 3.944 min), and p‐cymene (6.25%, RT = 7.320 min). Other identified compounds included α‐thujene (5.88%), camphene (4.25%), linalool (4.21%), caryophyllene (3.99%), and terpinene (2.65%). Similarly, *Pistacia atlantica* essential oil contained notable levels of β‐pinene (13.66%, RT = 4.821 min), limonene (11.48%, RT = 5.144 min), α‐pinene (7.41%, RT = 3.824 min), and p‐cymene (4.85%, RT = 7.314 min), along with linalool (3.99%), α‐thujene (3.98%), camphene (3.85%), caryophyllene (3.11%), and terpinene (2.04%). These results indicate that both oils are dominated by monoterpenes (α‐pinene, β‐pinene, limonene, p‐cymene) with smaller contributions from sesquiterpenes such as caryophyllene.

The predominance of α‐ and β‐pinene, limonene, and p‐cymene suggests strong biological relevance, as these monoterpenes are well documented for their antioxidant, antimicrobial, and anti‐inflammatory properties. For instance, α‐pinene and β‐pinene modulate oxidative stress by scavenging reactive oxygen species and enhancing endogenous antioxidant enzymes (Martins et al. 2019). Limonene is reported to exert anti‐inflammatory activity through inhibition of NF‐κB signaling, thereby reducing pro‐inflammatory cytokines (Kathem et al. 2024). Caryophyllene, a sesquiterpene, interacts with the CB2 receptor, contributing to analgesic and anti‐inflammatory responses (Jha et al. 2021). The synergistic presence of these compounds may explain the traditional medicinal applications of Boswellia and Pistacia gums.

These findings align with earlier reports. Koutsoudaki et al. (2005) identified α‐pinene, β‐pinene, β‐myrcene, β‐caryophyllene, and limonene as key constituents in *Pistacia lentiscus* essential oil, confirming the dominance of pinenes across Pistacia species. Ayub et al. (2023) also highlighted α‐pinene as the main compound (64.8%) in Pistacia sp. Similarly, Aziz et al. (2022) demonstrated that *Boswellia serrata* essential oil is rich in α‐pinene, accompanied by α‐thujene, trans‐verbenol, β‐thujone, and p‐cymene. More recently, Obiștioiu et al. (2023) reported α‐pinene (39.34%) and limonene (13.79%) as major components, consistent with our results. Older studies by Rao and Kaur (1989) and Mohammed et al. (2011) also documented a diverse terpene profile, further confirming the phytochemical complexity of frankincense oils.

The compositional similarity between frankincense and *Pistacia atlantica* oils, particularly in their shared abundance of α‐ and β‐pinene, limonene, and p‐cymene, highlights a conserved biosynthetic pathway of monoterpenes in resinous gums. These terpenoids are derived from the mevalonate and methylerythritol phosphate pathways, where geranyl pyrophosphate acts as a precursor for monoterpene synthesis (Pichersky and Raguso 2018). Such biosynthetic commonalities explain the overlapping aromatic and pharmacological properties of these resins.

Altogether, the phytochemical profiles of both essential oils underscore their therapeutic, aromatic, and industrial significance. Their high content of bioactive terpenes supports traditional uses in aromatherapy, perfumery, and natural medicine, while also presenting potential applications in modern pharmaceutical formulations.

### 3.4 Animal Experiment

#### 3.4.1 Impact of Functional Beverages on the Fecal Microbiota of Hypercholesterolemic Rats

**FIGURE 8:**
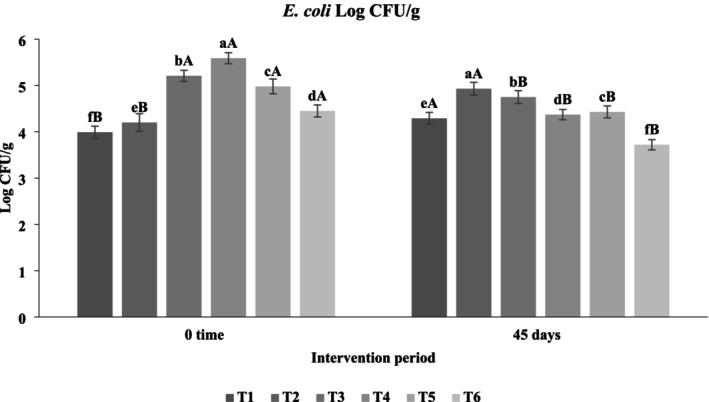
Logarithmic counts of *Escherichia coli* in feces of hypercholesterolemic rats. T1: (C−) fed a standard diet, T2: (C+) fed a standard diet plus 1% cholesterol, T3: Frankincense (F) fed a standard diet plus 1% cholesterol +frankincense raw beverage, T4: Frankincense Syn (FS) fed a standard diet plus 1% cholesterol+frankincense+1% FOS+ *Lactobacillus acidophilus* , T5: *Pistacia* (P) fed a standard diet plus 1% cholesterol+ *Pistacia atlantica* raw beverage, T6: *Pistacia Syn* (PS) fed a standard diet plus 1% cholesterol+ *Pistacia atlantica* +1% FOS+ *Lactobacillus acidophilus*. The lowercase letters indicate significant differences between samples within the same storage period, while the uppercase letters represent significant differences between storage periods for the same sample.

**FIGURE 9:**
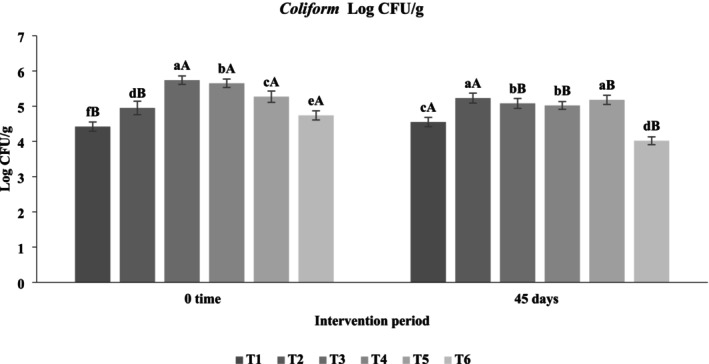
Logarithmic c*oliform* in the feces of rats with induced hypercholesterolemia. T1: (C−) ed a standard diet, T2: (C+) fed a standard diet plus 1% cholesterol, T3: Frankincense (F) fed a standard diet plus 1% cholesterol +frankincense raw beverage, T4: Frankincense Syn (FS) fed a standard diet plus 1% cholesterol+frankincense+1% FOS+ *Lactobacillus acidophilus* , T5: *Pistacia* (P) fed a standard diet plus 1% cholesterol+ *Pistacia atlantica* raw beverage, T6: *Pistacia Syn* (PS) fed a standard diet plus 1% cholesterol+ *Pistacia atlantica* +1% FOS+ *Lactobacillus acidophilus* . The lowercase letters indicate significant differences between samples within the same storage period, while the uppercase letters represent significant differences between storage periods for the same sample.

**FIGURE 10:**
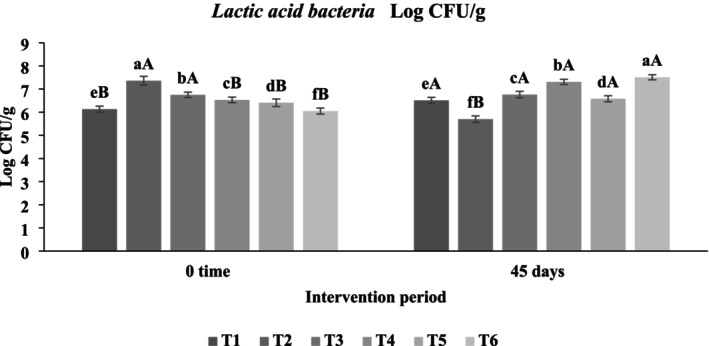
Logarithmic lactic acid bacteria in the feces of rats with induced hypercholesterolemia. T1: (C−) fed a standard diet, T2: (C+) fed a standard diet plus 1% cholesterol, T3: Frankincense (F) fed a standard diet plus 1% cholesterol +frankincense raw beverage, T4: Frankincense Syn (FS) fed a standard diet plus 1% cholesterol+frankincense+1% FOS+ *Lactobacillus acidophilus* , T5: *Pistacia* (P) fed a standard diet plus 1% cholesterol+ *Pistacia atlantica* raw beverage, T6: *Pistacia Syn* (PS) fed a standard diet plus 1% cholesterol+ *Pistacia atlantica* +1% FOS+ *Lactobacillus acidophilus* . The lowercase letters indicate significant differences between samples within the same storage period, while the uppercase letters represent significant differences between storage periods for the same sample.

The administration of functional beverages enriched with *Boswellia serrata* (frankincense) and *Pistacia atlantica* significantly modulated the fecal microbiota composition of hypercholesterolemic rats during the 45‐day intervention (Figures 8, 9, 10). The data revealed clear trends: (i) a reduction in pathogenic bacteria ( *E. coli* and coliforms), and (ii) a marked increase in beneficial lactic acid bacteria (LAB), with several differences reaching statistical significance (*p* < 0.05). Among the treatments, T6 ( *P. atlantica* combined with probiotics and prebiotics) demonstrated the most pronounced modulatory effects, exhibiting the highest LAB counts (7.51 log cfu/g) and the lowest *E. coli* (3.72 log cfu/g) and coliform levels (4.02 log cfu/g). In contrast, control groups C1 and C2 showed elevated *E. coli* levels (4.29 and 4.93 log cfu/g, respectively), highlighting the protective role of the functional beverages. Treatments T4 (frankincense with probiotics and prebiotics) and T3 ( *P. atlantica* with probiotics) also significantly increased LAB populations (7.31 and 6.76 log cfu/g, respectively) while reducing pathogenic bacteria compared to controls, though less effectively than T6. Interestingly, treatment T5 exhibited the highest coliform count (5.18 log cfu/g), suggesting that formulation type influences antibacterial efficacy. The antibacterial effects of *P. atlantica* can be mechanistically explained by its essential oil constituents α‐pinene, β‐pinene, myrcene, limonene, and terpineol, which disrupt bacterial cell membranes, leading to leakage of cytoplasmic contents and cell death (Dalvand et al. 2024). This explains the significant (*p* < 0.05) reductions in coliform and *E. coli* counts in T6 and T3 compared to controls. Likewise, frankincense contains boswellic acids with known antimicrobial and anti‐inflammatory properties, which likely contributed to the reductions in coliforms observed in T4, in agreement with Rovinaru and Pasarin (2020). The synergistic effects of Lactobacillus strains and fructooligosaccharides (FOS) further amplified microbial modulation. Prebiotics such as FOS selectively stimulate probiotic growth and metabolic activity, resulting in the production of short‐chain fatty acids (acetic, lactic, benzoic acids) and bacteriocin‐like substances that inhibit the proliferation of pathogenic bacteria (Tomar et al. 2015). This mechanistic pathway supports the significantly higher LAB counts (*p* < 0.05) observed in T6 and T4 compared with controls. Additionally, probiotic supplementation has been linked to improvements in lipid metabolism by lowering serum cholesterol and triglycerides while increasing HDL‐C, a benefit of particular relevance to hypercholesterolemic models (Hosseini et al. 2020). The current findings align with prior studies demonstrating the broad‐spectrum antibacterial efficacy of *P. atlantica* against both Gram‐negative and Gram‐positive bacteria (Rahman 2018; Ghalem and Mohamed 2009; Ismail et al. 2019). Similarly, Rovinaru and Pasarin (2020) reported reductions in *E. coli* with frankincense supplementation in rabbits, corroborating our observations in rats. Beyond microbial modulation, the integration of probiotic and prebiotic components is consistent with the concept of synbiotics, which are increasingly recognized for their ability to enhance gut health and improve host metabolic outcomes (Sivamaruthi et al. 2019). Collectively, these results indicate that functional beverages combining phytochemicals (frankincense or *P. atlantica* oils) with probiotics and prebiotics exert complementary mechanisms: (i) direct antimicrobial activity through membrane disruption, (ii) promotion of probiotic colonization and metabolic activity, and (iii) indirect benefits for host lipid metabolism. The statistical significance of these findings (*p* < 0.05) highlights the robustness of the effects, supporting the hypothesis that such functional beverages represent a promising dietary strategy for modulating gut microbiota and improving metabolic profiles in hypercholesterolemic conditions.

#### 3.4.2 Effects of Functional Beverages on Biochemical Blood Parameters in Rats With Induced Hypercholesterolemia

##### 3.4.2.1 Effect of Functional Beverages on Serum Triglyceride (TG) Levels

**FIGURE 11:**
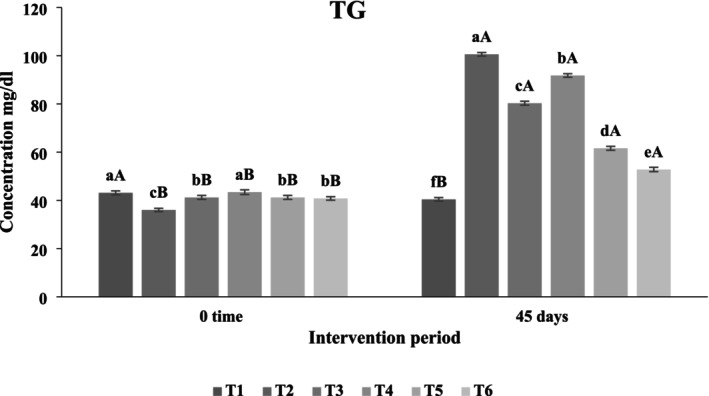
The effect of functional beverages on the triglyceride (TG) levels. T1: (C−) Fed a standard diet, T2: (C+) fed a standard diet plus 1% cholesterol, T3: Frankincense (F) fed a standard diet plus 1% cholesterol +frankincense raw beverage, T4: Frankincense Syn (FS) fed a standard diet plus 1% cholesterol+frankincense+1% FOS+ *Lactobacillus acidophilus* , T5: *Pistacia* (P) fed a standard diet plus 1% cholesterol+ *Pistacia atlantica* raw beverage, T6: *Pistacia Syn* (PS) fed a standard diet plus 1% cholesterol+ *Pistacia atlantica* +1% FOS+ *Lactobacillus acidophilus*. The lowercase letters indicate significant differences between samples within the same storage period, while the uppercase letters represent significant differences between storage periods for the same sample.

Figure 11 illustrates the impact of administering various functional beverages on serum triglyceride concentrations in hypercholesterolemic rats over a 45‐day period. Statistical analysis revealed significant differences (*p* < 0.05) among treatments. The fourth treatment group exhibited the highest triglyceride level (91.8 mg/dL), followed by the third treatment (80.3 mg/dL). Conversely, the fifth treatment group recorded a value of 61.6 mg/dL. Notably, the sixth treatment, which involved the administration of a synbiotic beverage comprising *Pistacia atlantica* , *Lactobacillus acidophilus* , and fructooligosaccharides (FOS), demonstrated the most pronounced reduction in serum TG levels, achieving 52.8 mg/dL. For comparison, the first and second control groups measured 40.4 mg/dL and 100.6 mg/dL, respectively.

##### 3.4.2.2 Effect of Functional Beverages on Serum High‐Density Lipoprotein (HDL) Levels

**FIGURE 12:**
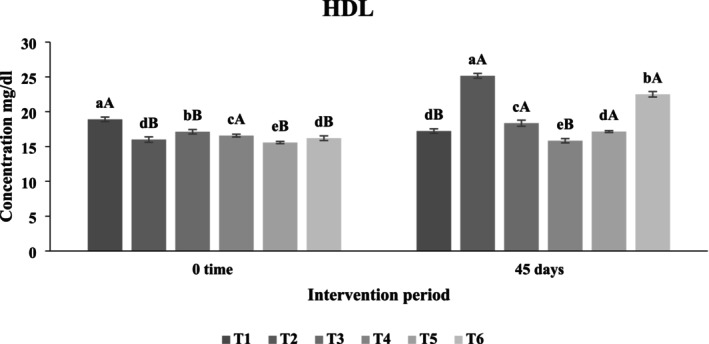
The effect of functional beverages on the high‐density lipoprotein (HDL) levels. T1: (C−) fed a standard diet, T2: (C+) fed a standard diet plus 1% cholesterol, T3: Frankincense (F) fed a standard diet plus 1% cholesterol +frankincense raw beverage, T4: Frankincense Syn (FS) fed a standard diet plus 1% cholesterol+frankincense+1% FOS+ *Lactobacillus acidophilus* , T5: *Pistacia* (P) fed a standard diet plus 1% cholesterol+ *Pistacia atlantica* raw beverage, T6: *Pistacia Syn* (PS) fed a standard diet plus 1% cholesterol+ *Pistacia atlantica* +1% FOS+ *Lactobacillus acidophilus*. The lowercase letters indicate significant differences between samples within the same storage period, while the uppercase letters represent significant differences between storage periods for the same sample.

The effect of functional beverage intake on serum HDL levels is shown in Figure 12. After 45 days, there were no statistically significant differences (*p* > 0.05) between the third and fifth treatment groups relative to the first control group, which had an HDL concentration of 17.22 mg/dL. The sixth treatment, a synergistic synbiotic formulation combining probiotics and prebiotics, exhibited the highest HDL concentration at 22.5 mg/dL, surpassing other groups. The third and fifth treatments resulted in HDL levels of 18.35 mg/dL and 17.15 mg/dL, respectively.

##### 3.4.2.3 Effect of Functional Beverages on Total Cholesterol (TC) Levels

**FIGURE 13:**
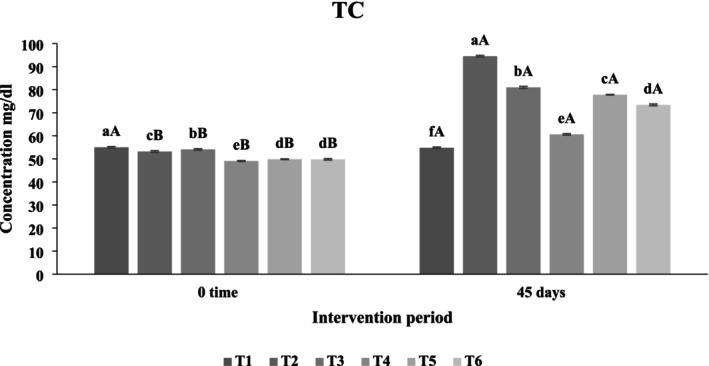
The effect of functional beverages on the total cholesterol (TC) levels. T1: (C−) fed a standard diet, T2: (C+) fed a standard diet plus 1% cholesterol, T3: Frankincense (F) fed a standard diet plus 1% cholesterol +frankincense raw beverage, T4: Frankincense Syn (FS) fed a standard diet plus 1% cholesterol+frankincense+1% FOS+ *Lactobacillus acidophilus* , T5: *Pistacia* (P) fed a standard diet plus 1% cholesterol+ *Pistacia atlantica* raw beverage, T6: *Pistacia Syn* (PS) fed a standard diet plus 1% cholesterol+ *Pistacia atlantica* +1% FOS+ *Lactobacillus acidophilus*. The lowercase letters indicate significant differences between samples within the same storage period, while the uppercase letters represent significant differences between storage periods for the same sample.

Figure 13 presents the effects of functional beverage administration on total cholesterol levels in hypercholesterolemic rats. After 45 days, significant differences (*p* < 0.05) were observed among treatments. The third treatment group recorded the highest mean TC level at 81 mg/dL, followed by the fifth treatment at 77.8 mg/dL. The fourth treatment, consisting of a pure frankincense beverage, yielded the lowest total cholesterol value (60.6 mg/dL), with the sixth synbiotic treatment ( *Pistacia atlantica* , *L. acidophilus* , and FOS) showing a moderate reduction to 73.4 mg/dL.

##### 3.4.2.4 Effect of Functional Beverages on Serum Low‐Density Lipoprotein (LDL) Levels

**FIGURE 14:**
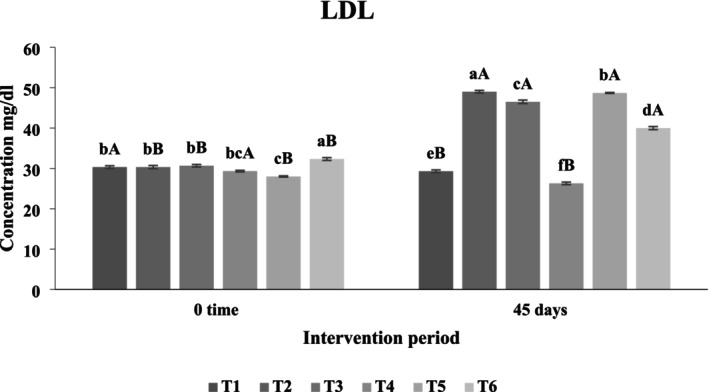
The effect of functional beverages on the low‐density lipoprotein (LDL) levels. T1: (C–) fed a standard diet, T2: (C+) fed a standard diet plus 1% cholesterol, T3: Frankincense (F) fed a standard diet plus 1% cholesterol +frankincense raw beverage, T4: Frankincense Syn (FS) fed a standard diet plus 1% cholesterol+frankincense+1% FOS+ *Lactobacillus acidophilus* , T5: *Pistacia* (P) fed a standard diet plus 1% cholesterol+ *Pistacia atlantica* raw beverage, T6: *Pistacia Syn* (PS) fed a standard diet plus 1% cholesterol+ *Pistacia atlantica* +1% FOS+ *Lactobacillus acidophilus*. The lowercase letters indicate significant differences between samples within the same storage period, while the uppercase letters represent significant differences between storage periods for the same sample.

As depicted in Figure 14, the administration of functional beverages influenced serum LDL levels after 45 days. No statistically significant differences (*p* > 0.05) were found among the sixth, fifth, and third treatment groups compared to the first and second control groups, which exhibited LDL concentrations of 29 mg/dL and 49 mg/dL, respectively. The fifth treatment recorded the highest LDL concentration at 48.7 mg/dL, closely followed by the third treatment at 46.5 mg/dL. The fourth treatment, featuring a synergistic beverage of *L. acidophilus* , FOS, and frankincense, achieved the lowest LDL level at 26.3 mg/dL, which was lower than that of the first control group (30.33 mg/dL). The sixth synbiotic treatment recorded an LDL concentration of 40 mg/dL.

##### 3.4.2.5 Effect of Functional Beverages on Very Low‐Density Lipoprotein (VLDL) Levels

**FIGURE 15:**
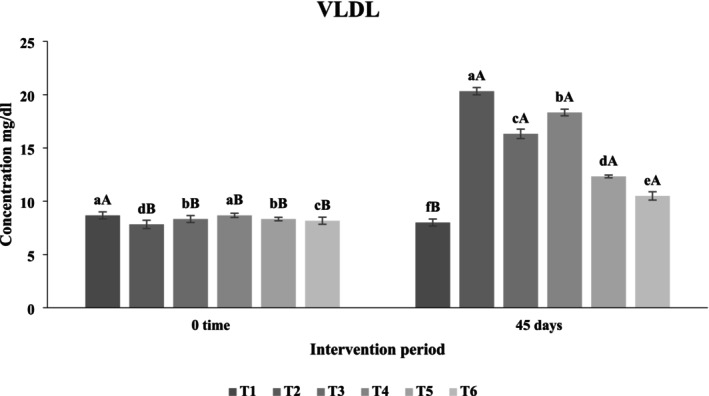
The effect of functional beverages on the very low‐density lipoprotein (VLDL) levels. T1: (C−) fed a standard diet, T2: (C+) fed a standard diet plus 1% cholesterol, T3: Frankincense (F) fed a standard diet plus 1% cholesterol +frankincense raw beverage, T4: Frankincense Syn (FS) fed a standard diet plus 1% cholesterol+frankincense+1% FOS+ *Lactobacillus acidophilus* , T5: *Pistacia* (P) fed a standard diet plus 1% cholesterol+ *Pistacia atlantica* raw beverage, T6: *Pistacia Syn* (PS) fed a standard diet plus 1% cholesterol+ *Pistacia atlantica* +1% FOS+ *Lactobacillus acidophilus*. The lowercase letters indicate significant differences between samples within the same storage period, while the uppercase letters represent significant differences between storage periods for the same sample.

Figure 15 shows the effects of functional beverages on serum VLDL levels in hypercholesterolemic rats. Significant differences (*p* < 0.05) were observed among treatments after 45 days. The fourth treatment group exhibited the highest VLDL concentration (18.33 mg/dL), whereas the sixth treatment, receiving the synbiotic beverage containing *Pistacia atlantica* , recorded the lowest level at 10.5 mg/dL. The fifth treatment, administering raw *Pistacia atlantica* beverage, showed a VLDL concentration of 12.33 mg/dL.

The results demonstrated that the sixth and fourth treatments significantly improved blood cholesterol levels compared to other treatments. This effect can be attributed to the inclusion of *Pistacia atlantica* gum, which, like many plant‐derived substances, is rich in phenolic compounds. These antioxidants are known to enhance endothelial function and inhibit the oxidation of low‐density lipoprotein (LDL) cholesterol, thereby reducing its arterial accumulation and subsequent cardiovascular risk (Chadzopulu et al. 2011). These findings align with prior studies highlighting the potent lipid‐lowering effects of *Pistacia atlantica* . The remarkable efficacy observed in the sixth treatment may be due to the natural mastic gum of *Pistacia*, which has been scientifically validated for its cholesterol‐absorbing properties, contributing to decreased risks of hypertension and myocardial infarction. Moreover, it has been shown to reduce triglycerides (TG) and total lipid levels systemically (Gomaa et al. 2019). Ghalavand et al. (2022) reported that *Pistacia atlantica* extract dose‐dependently lowered cholesterol, TG, and LDL‐C, while significantly increasing high‐density lipoprotein cholesterol (HDL‐C) compared to controls. Similarly, continuous administration of *Pistacia atlantica* extract for 3 weeks improved lipid profiles, attenuated oxidative stress, and modulated inflammatory processes by decreasing lipid peroxidation and enhancing total antioxidant capacity (Chadzopulu et al. 2011). The hypocholesterolemic effect observed in the fourth treatment is likely related to frankincense ( *Boswellia serrata* ), which has been documented to reduce blood cholesterol levels. Ahangarpour et al. (2014) demonstrated that administering *Boswellia serrata* (300 mg thrice daily for 6 weeks) significantly improved HDL, LDL, and total cholesterol levels in patients with type 2 diabetes. Additionally, Masoud et al. (2017) showed that rats fed a high‐fat diet supplemented with frankincense (300–400 mg/kg) exhibited significant improvements in lipid profiles, including reductions in total cholesterol, free fatty acids, TG, and LDL, along with increased HDL concentrations. The antioxidant components of frankincense mitigate oxidative stress, a critical factor in LDL oxidation and atherosclerotic plaque formation (Al‐Riyami et al. 2017; Santiago et al. 2010). Phenolic constituents such as beta‐pinene, limonene, and alpha‐pinene present in these natural products exhibit strong antioxidant properties and have been shown to reduce blood pressure, blood glucose, and lipid levels. These compounds effectively increase serum HDL‐C while decreasing TG, fasting blood glucose, LDL‐C, and improve glucose tolerance in obese murine models undergoing therapeutic interventions (Jing et al. 2013; Santos et al. 2022, 2023; Ghorbani et al. 2023). Furthermore, the addition of synbiotics significantly contributes to lipid profile improvement. Synbiotic formulations enhance intestinal health by modulating gut microbiota composition, thereby reducing plasma cholesterol levels. The presence of appropriate prebiotics fosters probiotic proliferation in the gastrointestinal tract, promoting a balanced microbial environment. Specifically, short‐chain fructans, such as fructo‐oligosaccharides, undergo fermentation by colonic bacteria to produce short‐chain fatty acids (SCFAs), which directly influence intestinal cholesterol metabolism by facilitating cholesterol uptake into bacterial cells and promoting its conversion to coprostanol—a mechanism central to reverse cholesterol transport. SCFAs have been demonstrated to reduce LDL‐C, very‐low‐density lipoprotein (VLDL), lipogenesis, and lipid storage in adipocytes and enterocytes (Altawari 2024). The cholesterol‐lowering effects of prebiotics may primarily result from two mechanisms: selective fermentation by intestinal microbiota generating SCFAs, and enhanced fecal excretion of cholesterol leading to reduced intestinal absorption (Hadi et al. 2020). Consistent with this, consumption of synbiotics has been associated with significant reductions in total cholesterol, TG, and LDL‐C levels, alongside increases in HDL‐C. These beneficial effects are more pronounced when synbiotics are administered as supplements over periods exceeding 8 weeks (He et al. 2017). Recent meta‐analyses confirm that probiotics significantly reduce total cholesterol, triglycerides, LDL‐C, systolic and diastolic blood pressure, and fasting blood glucose compared to placebo, while improving HDL quality (De Albuquerque et al. 2024).

#### 3.4.3 Probiotic Viability

The viability of *Lactobacillus acidophilus* was assessed in functional beverages fortified with *Boswellia sacra* (frankincense) and *Pistacia atlantica* during a 28‐day refrigerated storage period at 4°C. As shown in Table 7, the microbial counts in beverages containing *Boswellia sacra* exhibited a significant (*p* ≤ 0.05) decline over time, beginning immediately from Day 0. Notably, the synbiotic formulation (Fs), which combined probiotic and prebiotic components, demonstrated significantly higher *L. acidophilus* viability throughout the entire storage duration compared to the probiotic‐only formulation (Fl). At the end of the storage period (Day 28), Fs retained a viable count of 4.270 log CFU/mL, whereas Fl exhibited a markedly lower count of 2.103 log CFU/mL. These findings highlight the protective role of prebiotic inclusion in enhancing the stability and survival of probiotic bacteria during cold storage.

**TABLE 7:** Viability of *Lactobacillus acidophilus* (log CFU/mL) in *Boswellia sacra* beverages during cold storage at 4°C.

Treatment	Before Incubation	Day 0	Day 7	Day 14	Day 21	Day 28
Fl	7.815	6.852	4.345	4.105	2.695	2.103
Fs	7.753	8.123	7.623	6.344	5.363	4.270
LSD	N.S	0.4252	0.1835	0.1303	0.1429	0.2827

*Note:* Values are expressed in log CFU/mL. Significant differences were observed from Day 0 onward.

Abbreviations: Fl, probiotic only; Fs, synbiotic (probiotic + prebiotic); N.S, not significant at *p* > 0.05.

Table 8 presents the survival profile of *Lactobacillus acidophilus* in *Pistacia atlantica* ‐fortified beverages during storage. Both the probiotic‐only (Pl) and synbiotic (Ps) formulations demonstrated a gradual reduction in viable probiotic counts over time. Notably, the synbiotic beverage (Ps), enriched with prebiotics, exhibited enhanced stability and higher cell viability compared to the probiotic‐only beverage. After 28 days of storage, Ps maintained a viable count of 1.910 log CFU/mL, whereas Pl showed a lower survival level of 1.230 log CFU/mL. Although earlier sampling points (up to Day 21) revealed statistically significant differences (*p* < 0.05) favoring the synbiotic formulation, no significant difference (*p* > 0.05) was observed between Ps and Pl at Day 28. These results underscore the beneficial effect of prebiotic fortification in sustaining probiotic viability, thereby potentially extending the functional shelf life of probiotic beverages fortified with *Pistacia atlantica* .

**TABLE 8:** Viability of *Lactobacillus acidophilus* (log CFU/mL) in *Pistacia atlantica* Beverages During Cold Storage at 4°C.

Treatment	Before incubation	Day 0	Day 7	Day 14	Day 21	Day 28
Pl	7.791	7.236	5.212	3.910	2.110	1.230
Ps	7.763	8.881	7.214	5.670	3.230	1.910
LSD	N.S	0.1895	0.4423	0.701	0.722	N.S

*Note:* Values are expressed in log CFU/mL. Significant differences (*p* ≤ 0.05) were observed up to Day 21.

Abbreviations: N.S, not significant at *p* > 0.05; Pl, probiotic only; Ps, synbiotic (probiotic + prebiotic).

## 4 Conclusion

This study demonstrates that functional beverages enriched with *Pistacia atlantica* and *Boswellia serrata* extracts, particularly when combined with probiotics ( *Lactobacillus acidophilus* ) and prebiotics (FOS), exert significant beneficial effects on gut microbiota composition and lipid metabolism in hypercholesterolemic rats. The synbiotic formulation notably enhanced the growth of beneficial lactic acid bacteria while suppressing harmful bacteria such as *Escherichia coli* and coliforms, reflecting a robust antimicrobial activity likely attributed to bioactive compounds like alpha‐pinene and limonene. Furthermore, the synbiotic beverages improved lipid profiles by reducing triglycerides, total cholesterol, and VLDL levels, alongside elevating HDL cholesterol, which are key markers for cardiovascular health. The observed hypocholesterolemic effects are possibly mediated through antioxidant phenolic compounds and modulation of gut microbiota, including increased production of short‐chain fatty acids. Importantly, the synbiotic beverages maintained probiotic viability better over 28 days of refrigerated storage, underscoring the protective role of prebiotics. These findings suggest that such functional beverages could serve as a promising nutritional strategy for managing hypercholesterolemia and promoting intestinal health. Future clinical studies are warranted to validate these effects in humans and to explore underlying mechanisms in greater detail.

## Author Contributions

**Nawfal Alhelfi:** conceptualization (equal), data curation (equal), formal analysis (equal), methodology (equal), project administration (equal), software (equal), writing – original draft (equal), writing – review and editing (equal). **Zainab Tariq Yaseen Al‐Edany:** conceptualization (equal), data curation (equal), formal analysis (equal), methodology (equal), project administration (equal), software (equal), writing – original draft (equal), writing – review and editing (equal). **Ammar B. Altemimi:** conceptualization (equal), data curation (equal), formal analysis (equal), methodology (equal), project administration (equal), software (equal), writing – original draft (equal), writing – review and editing (equal). **Zheng Ruan:** conceptualization (equal), data curation (equal), formal analysis (equal), methodology (equal), project administration (equal), software (equal), writing – original draft (equal), writing – review and editing (equal). **Rawaa H. Tlay:** conceptualization (equal), data curation (equal), formal analysis (equal), methodology (equal), project administration (equal), software (equal), writing – original draft (equal), writing – review and editing (equal). **Tarek Gamal Abedelmaksoud:** conceptualization (equal), data curation (equal), formal analysis (equal), methodology (equal), project administration (equal), software (equal), writing – original draft (equal), writing – review and editing (equal).

## Ethics Statement

The research study was reviewed and approved by the Research Ethics Committee of the University of Basrah, College of Veterinary Medicine. The study received ethical approval under the approval number 70/37/2025, dated 22/4/2025. All ethical guidelines for research involving animals, as outlined by international standards and the University's policies, were strictly followed throughout the study.

## Consent

All authors have read and agreed to the published version of the manuscript. All authors read and approved the final manuscript.

## Conflicts of Interest

The authors declare no conflicts of interest.

## Data Availability

All data generated or analyzed during this study are included in this published article.
